# Supplementary material for: Acceptance and commitment therapy (ACT)-based programs for reducing symptoms of depression, anxiety and/or stress applied to the educational context: An exploratory review
Source: PLOS Ment Health. 2026 May 7;3(5):e0000234. doi: 10.1371/journal.pmen.0000234 (PMC13152172; doi:10.1371/journal.pmen.0000234)
Supplement: Attachment — Submitted filename: Response to Reviewers.pdf [file pmen.0000234.s004.pdf]

Bogotá, D.C., September 9, 2025

Dear reviewers,
PLOS Mental Health.

Subject: Verification of corrections to the article "Acceptance and Commitment Therapy (ACT)-based programs for reducing symptoms of depression, anxiety, and/or stress applied to the educational context: a scoping review."

In accordance with the comments sent by the journal reviewers, I would like to list the corrections made to the document:

1. 1. Reviewer number 1 points out that not all the data supporting the findings in the manuscript have been provided. However, the tables included present the relevant information that supports the characteristics of the programs. Although this information is summarized, the reader is provided with the criteria and particularities of each of the programs evaluated for the purpose of this article. Thus, when considering the tables as a whole, detailed information is available to support the objective of the manuscript.
2. Reviewer 1 points out the need to include a justification that shows how this review differs from previous reviews, thus providing an overview of what has already been done. In response to this comment, information was added on pages 4 and 5, highlighting that this review provides an overview of how programs are designed and implemented in the educational context. In this way, it not only complements previous reviews that have addressed ACT change mechanisms, but also integrates the analysis of the composition of interventions in order to assess whether the effectiveness of the results is also linked to the way in which programs are created and structured.
3. Reviewer 1 indicates that the end date of the literature search is not specified. However, on page 5, in the Method section, under the search process and procedure, both the start and end dates of the search are indicated.
4. Reviewer 1 suggests that, in the data extraction, the contribution of the reviewers in the search and selection of articles should be specified, and recommends that, if this has not been done, it should be mentioned in the limitations. In this regard, given that I am the sole author of this article, I have taken the observation into consideration and this characteristic has been incorporated into the limitations section.

5. Reviewer 1 points out that it is unclear why a considerable number of studies were excluded. In response to this observation, the absence of an exclusion criterion related to the lack of accessibility to the articles was identified. For this reason, this criterion was incorporated and, together with the PRISMA flow diagram, the studies that were excluded are detailed precisely.

6. Reviewer 2 highlights the importance of including the effect sizes of the reviewed studies. In response to this recommendation, wording was added to the Discussion section indicating the medium and large effect sizes evidenced in the articles, which allows for the effectiveness of interventions based on Acceptance and Commitment Therapy to be measured.

7. Reviewer 2 points out the importance of conducting a comparative analysis with other interventions, such as Cognitive Behavioral Therapy. In response to this observation, a paragraph was added to the Introduction section to provide greater clarity on the differences found in comparative studies, highlighting in particular the prevalence of research comparing Acceptance and Commitment Therapy with Cognitive Behavioral Therapy.

8. Reviewer 2 points out the need to analyze the barriers that may arise when implementing an intervention based on Acceptance and Commitment Therapy. In response to this observation, information was added to the Discussion section, as these barriers may influence the possibility of replicating these interventions in educational contexts.
