# Supplementary material for: Acceptance and commitment therapy (ACT)-based programs for reducing symptoms of depression, anxiety and/or stress applied to the educational context: An exploratory review
Source: PLOS Ment Health. 2026 May 7;3(5):e0000234. doi: 10.1371/journal.pmen.0000234 (PMC13152172; doi:10.1371/journal.pmen.0000234)
Supplement: Attachment — Submitted filename: Response_to_Reviewers_auresp_2.pdf [file pmen.0000234.s005.pdf]

Bogotá, D.C., 5 de febrero de 2026

Dear reviewers,
PLOS Mental Health.

Subject: Verification of corrections to the article "Acceptance and Commitment Therapy (ACT)-based programs for reducing symptoms of depression, anxiety, and/or stress applied to the educational context: a scoping review."

In accordance with the comments sent by the journal reviewers, I would like to list the corrections made to the document:

1. The Reviewer notes that, for greater clarity and transparency, it is necessary to indicate the exact date of the database search, since the manuscript only reported the date the article was written. In response to this observation, the timeframe has been expanded to include the period corresponding to the article search, the creation of the database, and the manuscript design, clearly specifying the start and end dates of each process.
2. The Reviewer also indicated the need to include, in the discussion section, a subsection that addresses the limitations of the study. Following this recommendation, this subsection was integrated into the discussion section.
3. The reviewer also notes the importance of describing how the effects reported in the included articles were evaluated. Therefore, a description of the data analysis used by the researchers was included, from which the results demonstrating the effect of the interventions on symptoms were obtained.

   Finally, in response to the recommendation to make explicit the evaluation of the quality of the studies, it is specified that this was carried out taking into account the methods used in each of the included investigations.
